# Synthesis of Gold Nanoparticles from Gold Coatings Recovered from E-Waste Processors

**DOI:** 10.3390/ma15207307

**Published:** 2022-10-19

**Authors:** Javier Su-Gallegos, Lorena Magallón-Cacho, Jeannete Ramírez-Aparicio, Edgar Borja-Arco

**Affiliations:** 1 Department of Theoretical Physics and Chemestry, Faculty of Chemistry, National Autonomous University of Mexico, Mexico City 04510, Mexico; 2 National Council of Science and Technology (CONACYT), Mexico City 03940, Mexico

**Keywords:** e-waste, gold recovery, nanoparticles, tetrachloroauric acid, Turkevich method

## Abstract

This work presents the synthesis of Au nanoparticles from gold coatings recovered from processor pins with minimal waste generation. The process consisted of four main steps: (1) physical recovery of pins, (2) recovery of gold coatings by acid digestion, (3) synthesis of HAuCl_4_ under mild conditions and, (4) synthesis of Au nanoparticles by the Turkevich method. The small dimensions of Au coatings allowed the synthesis of HAuCl_4_ with lower amounts of HCl_conc_ and HNO_3conc_ than those used with aqua regia. This method has significant advantages, such as lower NO_2(g)_ emission, easy post-treatment and purification, low synthesis cost and high yields. Gold nanoparticles synthesized from HAuCl_4_ were characterized by transmission electron microscopy (TEM) and UV-Vis spectroscopy. Size distribution analysis showed particles 14.23 nm in length and 12.05 nm in width, while absorption spectra showed a surface plasmon located at 523 nm; these characteristics were very similar to those observed with Au nanoparticles obtained with Aldrich’s reagent. It is suggested that recycling procedures can be improved by taking into account the size and shape of the metals to be recovered, thus introducing a new field of research known as hydronanometallurgy.

## 1. Introduction

Over the last decade, e-waste worldwide has been steadily increasing. Its negative impact on the environment has posed new challenges for waste management and current recycling processes [1,2,3,4]. Traditionally, this type of waste is processed by hydrometallurgy and pyrometallurgy [2,4,5]. However, these practices continue to produce toxic waste and the emission of harmful agents, which may endanger public and environmental health [4,6,7]. For all these reasons, the development of new recycling methodologies with environmental approaches has become one of the most studied topics in the world. Some of the main goals are: (1) reduce waste generation or reuse it to avoid landfilling, (2) optimize the collection system to reduce the environmental impact due to its transfer, (3) integrate a recycling chain with chemical and physical methods that optimize the process, (4) reduce the use of leaching agents or use less toxic reagents, and (5) optimize the current recycling processes [2,3,4]. These changes would minimize the environmental impact of waste and contribute to the United Nations Sustainable Development Goals (SDGs), such as “clean water and sanitation”, “sustainable cities and communities” and “responsible production and consumption” (goals 6, 11 and 12, respectively) [8].

Overall, recycling processes focus on concentrating the metals of interest (Au, Ag, Cu, Pd and others) in solution or in an ingot for selective separation [6,9,10,11]. Leaching agents such as thioureas, halides, cyanides and aqua regia are commonly used to separate the gold. Although they are considerably toxic with a significant environmental impact, their use is still justified by their yields [12,13,14].

Most research does not consider the size and shape of the Au to be recovered in the design of new methodologies [11,12,13,15,16,17,18,19]. However, other factors such as the size and shape of the gold to be recovered must be taken into account. These factors may be relevant because the chemical properties of gold change when it has smaller dimensions, becoming more reactive than in macroscopic dimension [20,21]. This property could be exploited for the design of new recycling methodologies. Understanding how these factors influence gold recovery could lead to improvements in recycling methods and a significant reduction in environmental impact.

Hydrometallurgical recycling methodologies recover gold in its oxidized form (HAuCl_4_), which is subsequently purified [9,12,22]. This acid has been produced for centuries by reacting metallic gold with aqua regia (a 3:1 mixture of concentrated hydrochloric and nitric acids, respectively); during this reaction, HNO_3_ oxidizes metallic gold to produce Au^3+^ ions, which will then form tetrachloroaurate (III) anions with the chloride ions. The continuous formation of the gold complex drives the reaction and generates H_2_O and NO as byproducts, the latter combining with ambient oxygen to generate NO_2_. Some studies have suggested that HNO_3_ may not be the only oxidizing agent in the reaction, as chlorine gas and nitrosyl chloride (produced during the reaction with aqua regia) can also oxidize small amounts of gold [23,24,25].

Although good yields are available, it has prompted the exploration of alternative synthesis methods, as the aqua regia method involves the use of concentrated acids and many steps for HAuCl_4_ purification, in addition to the environmental impact. For example, Shirin R. King et al. [26] propose a simple procedure using chlorine gas in a vessel with gold pellets and water (Equation (1)), where the authors report synthesis times ranging from 12 to 125 h depending on the temperature (room temperature, 50, 60 and 70 °C) and the size of gold pellets (spherical pellets and spherical pellets flattening at 1 mm thicknesses). The tetrachloroauric acid obtained with this method is stable and has a high purity, since the manganese used during the chlorine production process does not leach into the final HAuCl_4_ solution. However, some aspects must be taken into account: (1) the metallic gold used must have a high degree of purity, (2) the presence of chlorine gas implies special equipment for industrial use and (3) if the chlorine gas is synthesized in situ, more reagents and filters are needed to avoid contamination of HAuCl_4_.

2Au^0^_(s)_ + 3Cl_2(g)_ + 2HCl_(aq)_ → 2HAuCl_4(aq)_ (1)

Another example is reported by Masashi Hojo et al. [23,24] who dissolve gold plates (0.1 mm × 2 mm × 5 mm) and gold wires (0.1, 0.25, and 0.5 mm diameter) in a 2 mol L^−1^ HNO_3_ solution, to which they add different salts (LiCl, NaCl, KCl, MgCl_2_, CaCl_2_ and AlCl_3_) at different concentrations (from 0.1 mol L^−1^ to 9 mol L^−1^). The dissolution times of the plates and gold wires ranged from 35 h to 30 min depending on the reaction conditions and temperature used (15–80 °C). In addition, it is necessary to implement a purification system to obtain HAuCl_4_ with high purity, which is not desired. On the other hand, reaction times can be very long under certain conditions, resulting in low yields. Dissolution of gold using a “dilute aqua regia” solution (a mixture of HNO_3_ and HCl, both at 1.0 mol L^−1^) is also proposed. According to their results, this solution can dissolve a gold wire at 60 °C in 35 h, but when salts are added to the system, time is reduced. However, as in the previous case, the use of salts and long-lasting reactions are not desirable.

The main reason for finding green or better methodologies to synthesize HAuCl_4_ lies in the fact that this acid is used for the synthesis of gold nanoparticles, which have a wide range of applications such as the transport of therapeutic agents, photodynamic therapy, as sensors and probes, or in medical treatments. Therefore, the development of new synthesis routes and the use of different precursors are of great interest. However, the synthesis of gold nanoparticles depends on many factors such as pH, concentration, temperature and stabilizing agent, among others; for example, contamination of a pipette tip could add foreign material, which could lead to changes in the size and shape of the nanoparticles [20].

Although there are many methodologies for the synthesis of gold nanoparticles, this work focused only on the use of the Turkevich method, as it is a widely used methodology [20]. Nevertheless, the precursor (HAuCl_4_) obtained in this work could be used in other synthesis methodologies. Thus, this work shows a methodology for the synthesis of tetrachloroauric acid from gold coatings obtained from electronic waste processors, which is subsequently used for the synthesis of gold nanoparticles. First, a method is proposed to separate gold coatings from e-waste processors by acid digestion with HNO_3_. This strategy focuses on the separation of gold rather than its dissolution and subsequent selective chemical separation, so the method includes physical pretreatment of the processors to improve recovery, as well as the use of gas traps to eliminate the emission of toxic compounds. Secondly, the dissolution conditions for the synthesis of tetrachloroauric acid are studied. In this study, HCl was used as the complexing agent and HNO_3_ as the oxidizing agent. Although these acids are used in the procedure with aqua regia mentioned above, the amounts and concentrations used in this work represent an improvement in the gold oxidation process since they decrease the production of toxic agents and facilitate the purification of the products. Finally, gold nanoparticles were synthesized with HAuCl_4_ obtained from gold coatings using the Turkevich method.

## 2. Materials and Methods

### 2.1. Recovery of Pins from E-Waste Processors

Intel Pentium 4 processors from a local recycling center were used to recover the gold coatings. The pins were cut from the processor boards and then washed in an ultrasonic bath (Cole-Parmer Model 08895-21, Vernon Hills, IL, USA) for 15 min with deionized water (H_2_O_DI_, 2.0 × 10^−6^ Ω^−1^ cm^−1^ Meyer, Ciudad de México, Mexico) and isopropyl alcohol (≥99.5% Meyer, Ciudad de México, Mexico) at a 1:1 ratio. Finally, the pins were dried at room temperature.

Cu, Ni, Fe and Au were quantified by energy dispersive X-ray spectrometry (EDS) using a Bruker-Quantax 200 energy dispersive x-ray spectrometer with a FE-SEM S-5500 microscope (Hitachi, London, UK). Three components of the pins were examined: the base, the coating and the inner body (using a colloidal silver sample preparation for the latter).

The morphology of the pins was characterized by scanning electron microscopy (SEM) using a JEOL-5600LV microscope (Japan Electron Optics Laboratory, Tokyo, Japan) at 20 kV, with secondary electrons, spot size of 500 and Working Distance (WD) of 8.5 mm.

Cu, Ni, Fe and Au were also quantified by microwave plasma atomic emission spectrometry (MP-AES) on an MP-AES 420 spectrometer (Agilent, Santa Clara, CA, USA) with a Multi-wave PRO microwave oven (Anton Paar, NSW, AU). For this purpose, 478 pins were dissolved in 10 mL of aqua regia (36.5–38.0% HCl_conc_, 68.0–70.0% HNO_3conc_, both Meyer, Ciudad de México, Mexico).

### 2.2. Recovery of Gold Coatings from Pins

**Figure 1:**
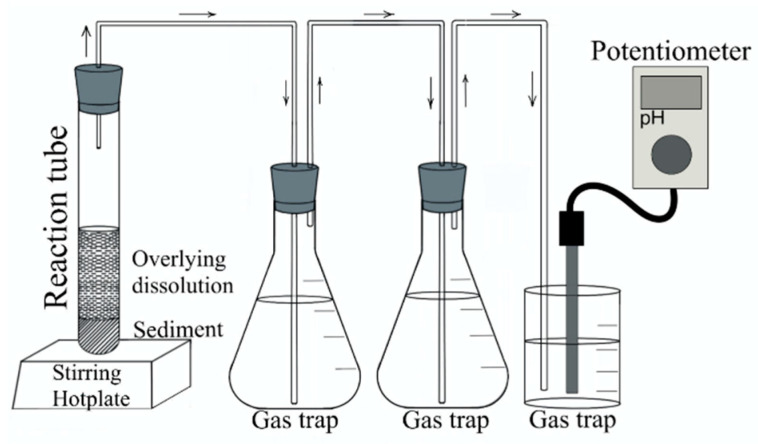
Experimental setup for the recovery of gold coatings from e-waste processor pins.

Gold coatings from three different batches of processors (each with 1434 pins which are equivalent to three processors) were separated by acid digestion with HNO_3conc_ using the experimental setup showed in Figure 1. The mass of the pins (uncertainties are indicated in parentheses) used for batches 1, 2, and 3 were 2.5119 (0.0004) g, 2.5392 (0.0004) g and 2.6118 (0.0002) g, respectively [27]. The volume of HNO_3conc_ was calculated using Equation (2), under the assumption that the pins were pure Cu. The reaction was started by placing the reaction tube on a hotplate stirrer (Cole-Parmer model JZ-03407-10, Vernon Hills, IL, USA) at 80 °C with three gas traps containing 50 mL of 50% *v*/*v* hydrogen peroxide (29.0–32.0% H_2_O_2_, High Purity, Ciudad de México, Mexico) in water [28,29,30]. Once the reaction was started, the hotplate was turned off and only agitation was maintained. To ensure the proper functioning of the gas traps, the initial and final pH was measured throughout the procedure, using a ROSS Ultra pH/ATC Triode (Thermo scientific model Orion 8107UWMMD, Waltham, MA, USA) and an Orion star potentiometer (Thermo scientific model A325, Waltham, MA, USA).

For Batch 1, a total of 9 mL of HNO_3conc_ was used by adding it 1 mL at a time into the reaction tube. Once the reaction was completed, 8 mL of H_2_O_DI_ was added (also 1 mL at a time) to improve the solubility of the products. While for Batch 2, a total of 8 mL of HNO_3conc_ was added into the reaction tube in three portions: (1) 5 mL at the beginning of the reaction, (2) 1 mL at the middle and (3) 2 mL at the end. In contrast to the first batch, in which water was added at the end of the reaction, for Batch 2, a total of 2 mL of H_2_O_DI_ was added halfway through the reaction. A lower amount of HNO_3conc_ was used for this second batch because it was previously observed that the reaction had already finished before the last milliliter of HNO_3conc_ was added (i.e., no NO_2(g)_ evolution was observed). For Batch 3, only 10 mL of HNO_3conc_ were added from the beginning of the reaction and no H_2_O_DI_ was added.

After completion of the acid digestion, the gold coatings were separated from the overlying dissolution with a pipette and rinsed with deionized water. The SnO_2_ formed (Equation (3)) and some plastic remainders were filtered and separated, which were subsequently weighed. Then, NaOH pellets (≥98% Sigma-Aldrich, St. Louis, MO, USA) were added to the filtered solution to generate the corresponding Cu(OH)_2_ according to Equation (4), which was finally heated to obtain the corresponding CuO according to Equation (5). This oxide was filtered and weighed. All solids obtained (Au coating, SnO_2_ and CuO) were characterized by X-ray Diffraction (XRD) using a Bruker AXS-D8 Advance Davinci X-ray diffractometer Theta-Theta configuration (Bruker Mexicana, Ciudad de México, Mexico) and SEM (Hitachi FE-SEM S-5500 microscope).

Cu_(s)_ + 4HNO_3(aq)_ → Cu(NO_3_)_2(aq)_ + 2NO_2(g)_ +2H_2_O_(l)_ (2)

Sn_(s)_ + 4HNO_3(aq)_ → SnO_2(s)_ + 4NO_2(g)_ + 2H_2_O_(l)_ (3)

Cu(NO_3_)_2(aq)_ + 2NaOH_(s)_ → Cu(OH)_2(aq)_ + 2NaNO_3(aq)_ (4)

Cu(OH)_2(aq)_ + heat → CuO_(s)_ + H_2_O_(aq)_ (5)

Another batch with three processors was treated similarly, but now only the remaining solutions after nitric acid digestion, i.e., the one with the mixture of Cu(NO_3_)_2_ and SnO_2_, and that obtained after CuO formation, were characterized by MP-AES to quantify the remaining Cu, Ni and Fe ions.

### 2.3. Synthesis of Tetrachoroauric Acid (HAuCl_4_)

The synthesis of HAuCl_4_ was carried out in duplicate on a hotplate stirrer at 125 °C in a borosilicate glass tube covered with a stopper by reacting 2 mg of Au coatings with 10 mL of 10% *v*/*v* HCl (1.21 mol L^−1^) and 50 μL of HNO_3conc_ (final concentration 0.08 mol L^−1^). For each synthesis, a control tube with the reaction media devoid of Au coatings was used. The solutions obtained were characterized by MP-AES, cyclic voltammetry (CV) using an AFP2 Bipotentiostat (PINE Wavedriver, Durham, NC, USA) and by UV-Vis with a calibration curve (CARY UV-Vis-NIR Spectrophotometer, model 5E, Ciudad de México, Mexico).

CV was performed in a cell with a three-electrode configuration: saturated Calomel electrode (Radiometer analytical, type XR100, VC, FR) as reference electrode; graphite rod as counter electrode; and glassy carbon disk (PINE modelAFE3T050GC, A = 0.1964 cm^2^, Durham, NC, USA) as work electrode. Potential sweeps were performed between −0.27 and 1.1 V vs. SCE at 50 mV s^−1^. To identify the electrochemical signals in the cyclic voltammograms, three 1 mM HAuCl_4_ solutions were prepared with Aldrich’s reagent (HAuCl_4_⋅3H_2_O, 99.9%, Sigma-Aldrich, MO, USA) at pH = 0.5, adjusted with HCl. To the first one, 7 aliquots of 200 µL of a 1 mM CuCl_2_ solution (CuCl_2_⋅2H_2_O, 98% Fisher Scientific, Waltham, MA, USA) at pH = 3.09 were added; to the second one, 2 aliquots of 200 µL of a 1 mM NiCl_2_ solution (NiCl_2_⋅6H_2_O, 90–100% Mallinckrodt Baker, DE) at pH = 3.64 were added; and to the latter, 5 aliquots of 200 µL and then 14 aliquots of 0.5 mL of a 1 mM FeCl_3_ solution (FeCl_3_⋅6H2O, 97% Sigma-Aldrich, St. Louis, MO, USA) at pH = 2.66 were added. In all cases, 8 mL of HAuCl_4_ was used and cyclic voltammetry was performed between each added aliquot, in addition to measuring the final pH in each solution.

### 2.4. Synthesis of Gold Nanoparticles

Once HAuCl_4_ was synthesized from gold coatings, the solution was evaporated to approximately 1 mL, and then 10 mL of H_2_O_DI_ was added, the evaporation process was repeated in triplicate, and in this last step the 1 mL of HAuCl_4_ obtained was transferred to a 10 mL volumetric flask and diluted to volume with H_2_O_DI_. Au, Ni, Fe and Cu were quantified by MPAES before and after each evaporation cycle. From the latter solution, gold nanoparticles were synthesized by the Turkevich method by mixing 5 mL of the HAuCl_4_ solution with 0.5 mL of 38.8 mM sodium citrate (C_6_H_5_Na_3_O_7_⋅2H_2_O, 100.1%, J.T. Baker, QC, Canada), and the solution was filtered through 7µm filter paper (Whatman grade 52, UK). As a comparison, gold nanoparticles were synthesized with Aldrich HAuCl_4_ reagent by mixing 250 mL of 1 mM HAuCl_4_ (also evaporated in triplicate) with 25 mL of 38.8 mM sodium citrate.

Both batches were characterized by TEM (Jeol model JEM2010 FEG, Tokyo, Japan) and UV-Vis spectroscopy to obtain the particle size distribution, and the absorption spectra to find the Localized Surface Plasmon (LSP).

## 3. Results and Discussion

### 3.1. Pin Recovery from E-Waste Processors

**Figure 2:**
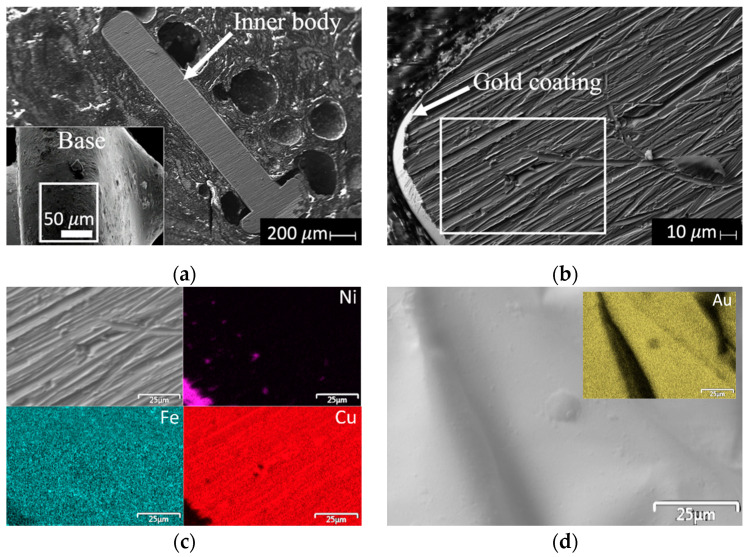
Scanning electron micrographs of: (**a**) inner body and base of a pin at 40× and 500× magnification, respectively; (**b**) inner body at 500× magnification (**c**) elemental maps of a section close to the coating inside the inner body of the pin; and (**d**) elemental map of the gold coating. The white box on the right indicates the area where elemental mapping was performed, and the box on the left shows where the chemical analysis of the base was performed.

Figure 2 shows SEM images of the inner body and base of a single pin, as well as elemental maps of the gold coating and a near-coated section inside the inner body of the pin. The inner body of the pins is composed of a homogeneous Cu/Fe phase coated with Ni. This composition becomes evident in Figure 2c, where the area close to the coating shows an isolated zone of Ni (purple map), while the areas away from this point show a mixture of Cu and Fe (red and blue maps, respectively). In addition, Figure 2d shows the elemental map obtained from a gold coating after the separation process. In this case, Au is the only element present in this part of the pin.

The presence of Fe and Cu in the inner body was expected, as the pins exhibited magnetism and reacted with HNO_3conc_ to produce a green-colored solution at the beginning and a blue-colored solution at the end of the reaction, which are related to the presence of Ni^2+^ and Cu^2+^ ions, respectively. Although there are technical reports on the processors, they may not include all the information on the elements present in these devices. For example, for Intel Pentium 4 processors, the technical report mentions the presence of Au, Ni and Cu, but there is no information on the presence of Fe [31].

Table 1 shows the EDS results with standard deviations in parentheses for the inner body, gold coating and pin base. The results obtained inside the inner body correspond to three different acquisition zones (top, middle and bottom zones in Figure 2a). The inner body of the pin contains 96.05% of Cu, while Ni (1.72%) and Fe (2.23%) are present in lower amounts. For the gold coating, only one signal associated with Au was detected, while only Sn was detected at the base.

On the other hand, the MP-AES results of the digestion in aqua regia of 478 pins are shown in Table 2. It can be observed that only the wt% Cu is close to that obtained by EDS (Table 1), and that Ni and Fe still represent approximately 5% of the total mass of the pins (excluding Sn). Furthermore, these results show that the quantity of Au represents only 0.9% of the total mass. Considering the manufacturer’s pin geometry information (r = 0.0306 cm and h = 0.1381 cm), and the mass of Au reported in Table 2, the thickness of the gold coatings was estimated to be 250 nm. This thickness is outside the 100-nm limit typically considered for the nanoscale. Therefore, a difference in their chemical properties would be expected.

With this information, an acid digestion using a low volume of HNO_3conc_ was proposed, since all the elements except Au react with this acid, which would favor the coating separation procedure. The difference between the method proposed in this work and the hydrometallurgical and pyrometallurgical methods lies in the fact that our method focuses on the direct separation of gold coatings, while in the other two methods the gold is dissolved or melted with other elements to be selectively separated, which directly affects the purity of the Au obtained and the yield of the process.

### 3.2. Recovery of Gold Coatings from Pins

**Figure 3:**
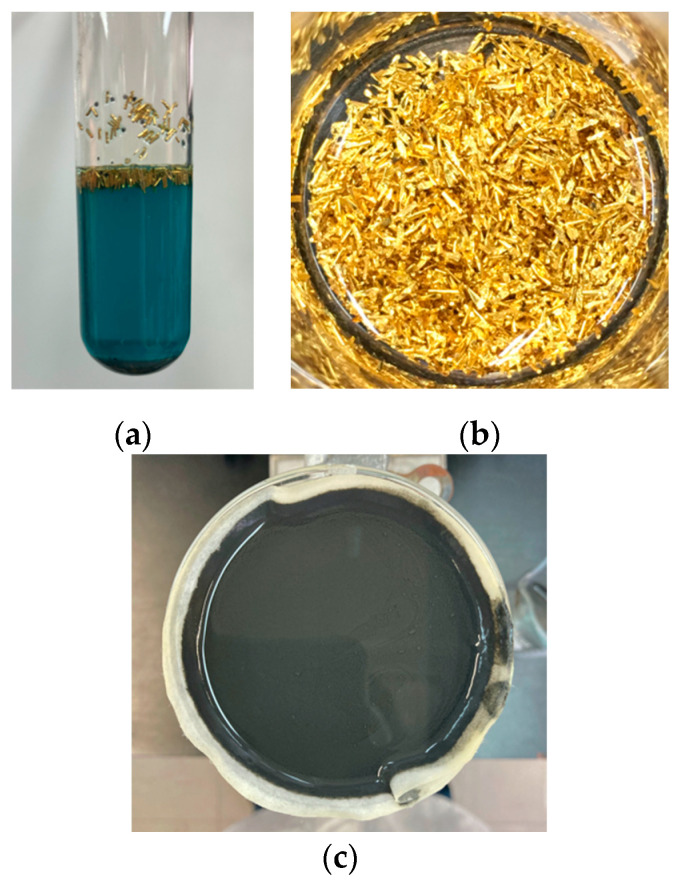
(**a**) Reaction tube after acid digestion with HNO_3conc_, (**b**) recovered gold coatings and (**c**) CuO filtration after complexing and precipitation reactions.

Figure 3a shows the reaction tube with the formation of Cu(NO_3_)_2_, Ni(NO_3_)_2_ and Fe(NO_3_)_3_ (blue solution), as well as SnO_2_ (gray precipitate at the bottom of the tube) and gold coatings (at the top), after acid digestion of the pins. Since the amounts of Ni and Fe are much lower than those of Cu, the solution was assumed to be composed of Cu(NO_3_)_2_ with a purity of 95% (Table 2). Similar behavior was observed in all three batches, allowing the gold coatings to be separated quickly and easily (Figure 3b). Au coatings and SnO_2_ were weighed and stored, while the Cu(NO_3_)_2_ was reacted with NaOH under heat to produce the corresponding CuO (Figure 3c). It was decided to obtain CuO to calculate the mass efficiency of the process. The solution with Cu(NO_3_)_2_, Ni(NO_3_)_2_ and Fe(NO_3_)_3_ obtained after acid digestion could be treated by another method to separate and recover Cu, Ni and Fe.

On the other hand, pH was observed to decrease in each of the NO_2_(g) traps. For each of the batches, the gas traps started at pH = 3.14 and at the end of the reaction the measured values were 0.85, 2.43 and 3.06 for the first, second and third traps, respectively, meaning that the traps reacted with NO_2_(g) to produce HNO_3_. The pH of the first trap decreased significantly, which is not surprising since it is the first to receive NO_2_(g). To confirm the presence of HNO_3_ (according to Equation (2)), a test was performed with a Cu wire and an aliquot with the solution of the first trap, obtaining Cu(NO)_3_ as reaction product.

**Figure 4:**
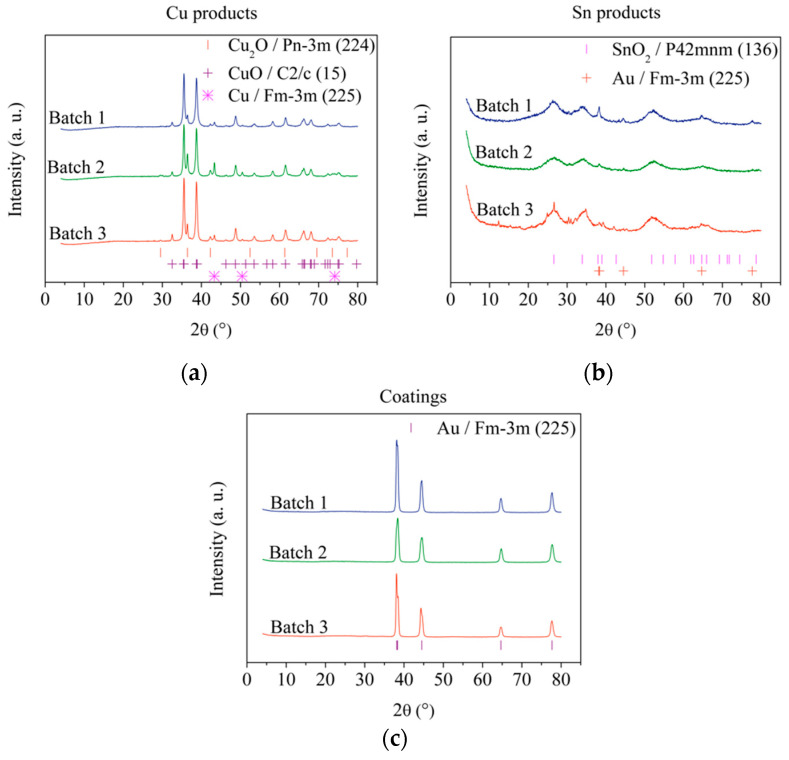
X-ray diffraction patterns of (**a**) Cu products (JCPDS files: 04-0836, 05-0667 and 48-1548 for Cu, Cu_2_O and CuO, respectively), (**b**) Sn products (JCPDS file: 41-1445 for SnO_2_) and (**c**) Au coatings recovered from batches 1, 2 and 3 (JCPDS file: 04-0836 for Au).

The products obtained during the gold recovery process were analyzed by X-ray diffraction; thus, Figure 4 shows the X-ray diffraction patterns of CuO, SnO_2_ and the Au coatings recovered from batches 1, 2 and 3, as well as the Bragg positions of the phases found. All X-ray patterns show similar profiles and intensities.

**Figure 5:**
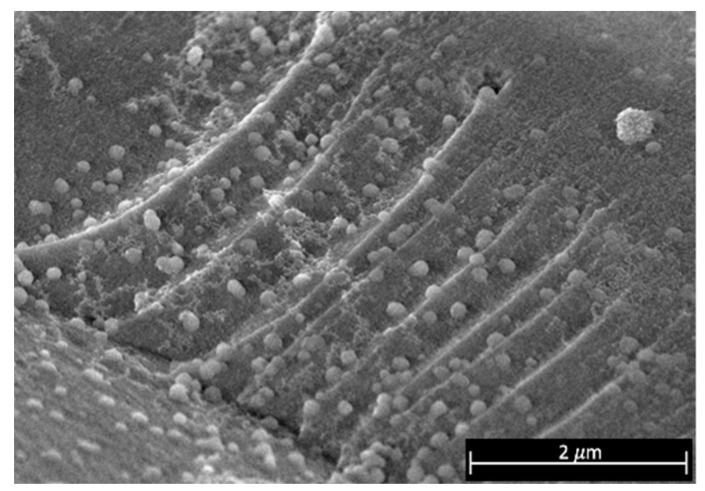
Scanning electron micrograph of SnO_2_ with Au particles on its surface.

The phases found in Figure 4a correspond to Cu, Cu_2_O and CuO. For efficiency calculations, it can be assumed that this product is composed entirely of CuO, since the CuO signals are more intense compared to Cu and Cu_2_O signals. No signals associated with any Ni or Fe phase were found due to the low amount of these elements. On the other hand, SnO_2_ and Au phases are observed in Figure 4b; according to morphological analysis (Figure 5), these Au particles are supported on SnO_2_. Due to the shape and size of the Au coatings, the support of the gold particles on SnO_2_ could be explained by the oxidation of a small portion of the Au coatings when HNO_3conc_ is added during their recovery, which is subsequently reduced when Sn is oxidized and finally deposited on the formed SnO_2_. Thus, for efficiency estimates, the precipitate was assumed to be 100% SnO_2_. Finally, the diffractograms of the gold coatings are shown in Figure 4c, where only signals associated with Au crystalline phases are observed, thus confirming the results obtained by EDS (Table 2) and indicating that the coatings consist only of Au with a high degree of purity.

Table 3 summarizes the masses of Au, Cu and Sn recovered from the pins, as well as the batch efficiencies based on the recovered masses and the results obtained by MP-AES, along with their associated uncertainties in parentheses [27]. Batch 1 shows the highest total mass (mT) and MP-AES efficiencies (96.8 and 89.0%, respectively), meaning that adding H_2_O_DI_ to the reaction medium (in an amount similar to that of HNO_3conc_) improves the yield, which could be due to an improvement in the solubility of Cu(NO_3_)_2_ (Equation (2)). Thus, for the other batches with lower H_2_O_DI_ volumes, the efficiencies were between 73–82%.

MP-AES analysis of a second sample (treated as Batch 1) shows that, after acid digestion, the mass of Cu, Ni and Fe is similar to that reported in the pretreatment section (Table 2 and Table 3). Thus, the procedure was able to dissolve approximately 99% of the pins (the other 1% corresponds to Au). Moreover, MP-AES analysis of the solution after complexation and oxidation shows the presence of Cu and Fe in small amounts (0.05341 and 0.00126 mg, respectively), meaning that the conditions for these reactions should be optimized. Based on these results, it can be established that the methodology fulfills the purpose of not generating waste during the separation of the gold coatings (which does not occur in most of the separation procedures mentioned above). In addition, the byproducts obtained can be treated to recover the original elements (Fe, Cu, Ni and Sn), while the solutions used in the gas traps can be treated to recover the HNO_3_ again.

The methodology used in this work could be optimized by evaluating the separation of gold coatings at different concentrations, times, volumes and temperatures, which would improve costs and reduce the environmental impact.

### 3.3. Synthesis of Tetrachloroauric Acid (HAuCl_4_)

**Figure 6:**
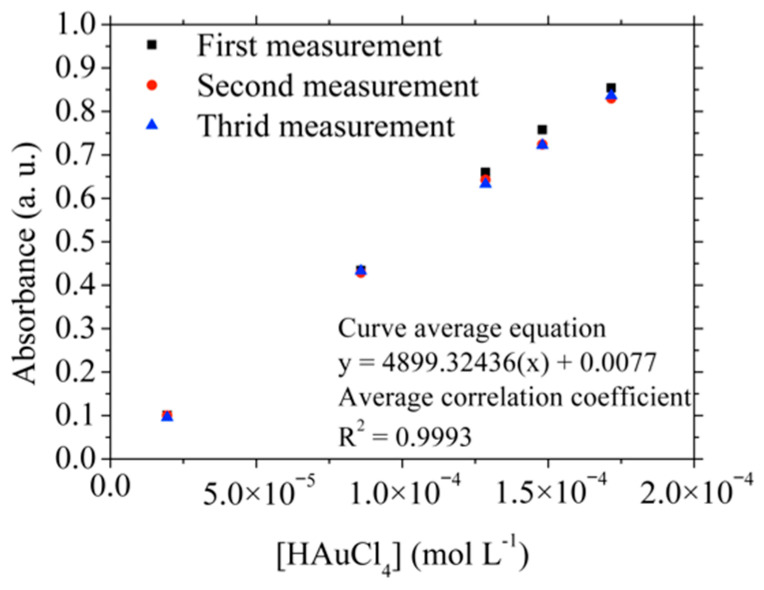
UV-Vis calibration curves between 310 and 320 nm.

Figure 6 shows the UV-Vis calibration curves obtained between 310 and 320 nm in triplicate, which were used to determine the concentration of the two synthesized HAuCl_4_ samples (labeled 1.1 and 1.2, respectively). These results were compared with the chemical analysis by MP-AES. In this way, Table 4 shows the results obtained from the calibration curve with their uncertainties in parenthesis [27] and the chemical analysis obtained by MP-AES. When comparing the Au concentrations obtained by UV-VIS and MP-AES, values very close to each other are observed, as is a complete dissolution of Au coatings in both samples, i.e., the percentages of dissolved Au are close to 100%.

The ability of the HCl/HNO_3_ mixture used in this work to dissolve Au coatings at much lower concentrations than that of aqua regia could be explained by taking into account its thickness. Thus, the estimated thickness of the gold coating was 250 nm, which cannot be considered at the nanoscale because the sample must be smaller than 100 nm; however, the results obtained in this work indicate that the dimensions of the coatings are small enough to affect the chemical properties of Au. These effects may be due to a low concentration of near-neighboring Au atoms at the edges, allowing gold atoms to have greater freedom to vibrate and oxidize, which confers to them significant chemical reactivity [21]. According to the different conditions for Au dissolution in the works shown above, none of them have focused on the effect of the size of their Au samples, which would imply one more variable to be contemplated.

The results obtained by MP-AES shown in Table 4 indicate that HAuCl_4_ synthesized by the conventional method has a purity (2–6 ppm trace metals) similar to that of Aldrich reagent (trace metals ≤ 1000 ppm, Product number: 520918, Batch: MKCJ4933). The synthesized HAuCl_4_ could also contain traces of Fe and other undetected elements (from water, HCl and HNO_3_ used during synthesis). However, even if all trace amounts present are considered, the number of traces will not exceed 1000 ppm.

**Figure 7:**
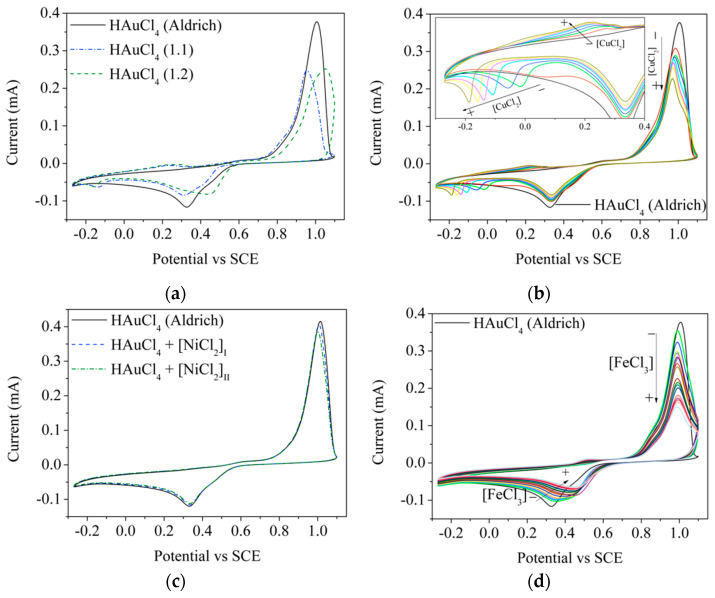
Cyclic voltammograms of (**a**) HAuCl_4_ synthesized compared to Aldrich’s reagent (pH_1.1_ = 0.60, pH_1.2_ = 0.78 and pH_Aldrich_ = 0.5), (**b**) Aldrich HAuCl_4_ with and without the presence of CuCl_2_ (pH = 0.69), (**c**) Aldrich HAuCl_4_ with and without the presence of NiCl_2_ (pH = 0.74) and (**d**) Aldrich HAuCl_4_ with and without the presence of FeCl_3_ (pH = 0.77).

Since the presence of Fe could not be determined by MP-AES analysis, the electrochemical technique of cyclic voltammetry was used for its determination. In this way, Figure 7 shows the cyclic voltammograms of different 1 mM HAuCl_4_ solutions used: from Aldrich (black solid lines) in the absence and presence of different salts (CuCl_2_, NiCl_2_ and FeCl_3_); and HAuCl_4_ synthesized (blue dotted line for 1.1, and green dotted line for 1.2).

Two peaks at 1.00 and 0.325 V/SCE are observed for all Aldrich HAuCl_4_ solutions, which are affected by the presence of CuCl_2_ and FeCl_3_ (Figure 7b,d, respectively), while the presence of NiCl_2_ does not affect these peaks (Figure 7c). The presence of CuCl_2_ (Figure 7b) results in a decrease in the currents for both peaks as the salt concentration increases, and another peak is observed between −0.2 and 0.25 V/SCE; these shift to more negative potentials as the CuCl_2_ concentration increases. A similar behavior is observed in the presence of FeCl_3_ (Figure 7d), i.e., as the concentration of the iron salt increases the currents in both peaks decrease, in addition to the peak at 0.325 V/SCE shifting towards more positive potentials.

Thus, for HAuCl_4_ 1.1 and 1.2, the presence of Cu is confirmed by the peak between −0.2 and 0 V/SCE, while the presence of Fe is observed only for HAuCl_4_ 1.2, as the peak at 0.325 V/SCE is shifted towards more positive potentials (0.47 V/SCE) (behavior observed in Figure 7b).

### 3.4. Synthesis of Gold Nanoparticles by the Turkevich Method

Table 5 shows the MP-AES results of the HAuCl_4_ solution before and after the evaporation cycles. It can be observed that the evaporation cycles do not contribute to improving the purity of the solution; in fact, these cycles did the opposite since the amounts of Fe and Cu increased while the concentration of HAuCl_4_ decreased by 41%.

**Figure 8:**
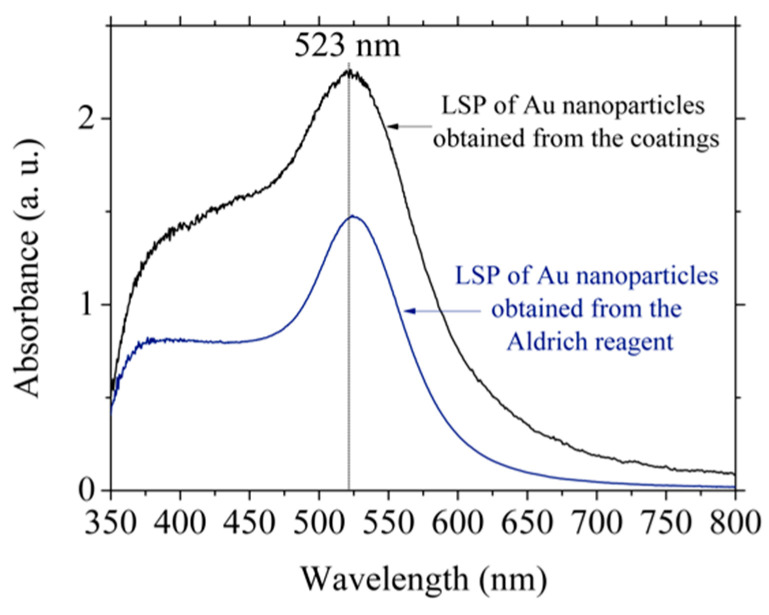
Absorption spectra of Au nanoparticles obtained from the coatings (black line) and from Aldrich’s reagent (blue line).

Gold nanoparticles were synthesized by the Turkevich method using HAuCl_4_ from Aldrich and that obtained after evaporation cycles. In this way, two wine-colored solutions were obtained at the end of synthesis, which confirmed the presence of Au nanoparticles. Figure 8 shows the absorption spectra of both solutions; the presence of a maximum at 523 nm related to the LSP can be observed in both batches. The absorbance recorded for Au nanoparticles obtained from the coatings was higher than those obtained from Aldrich’s reagent. Based on the position of the peaks, a particle size between 15 and 20 nm was assumed [32], which was congruent with the synthesis methodology.

**Figure 9:**
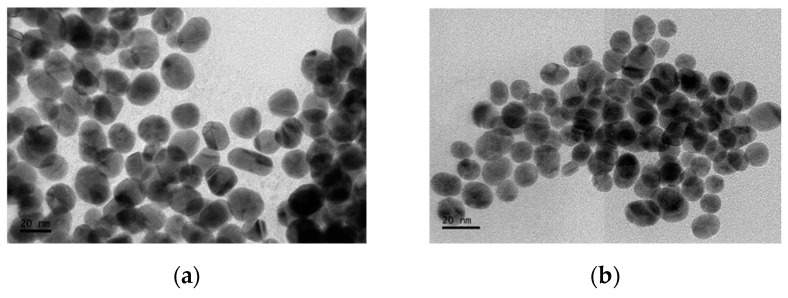

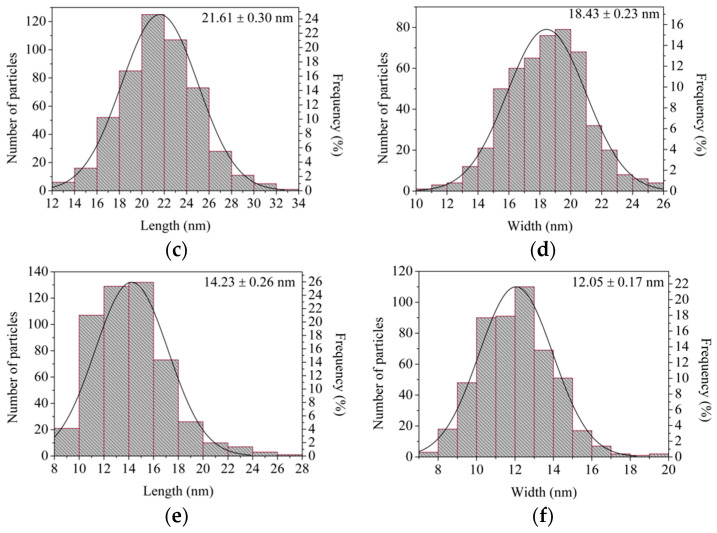
Transmission electron micrograph of gold nanoparticles obtained from (**a**) Aldrich reagent and (**b**) gold coatings; (**c**) length and (**d**) width of Au nanoparticles from Aldrich, and (**e**) length and (**f**) width of Au nanoparticles obtained from coatings.

Figure 9 shows the transmission electron micrographs of Au nanoparticles obtained from Aldrich’s reagent and those obtained from gold coatings, as well as size distribution analysis (509 particles were measured for each using ImageJ software for the analysis [33]). The latter showed that the Aldrich nanoparticles (length = 21.61 ± 0.30 nm, width = 18.43 ± 0.23 nm, and equivalent circular diameter = 20.30 ± 0.24 nm) were larger in size than the Au nanoparticles obtained from the coatings (length = 14.23 ± 0.26 nm, width = 12.05 ± 0.17 nm, and equivalent circular diameter = 13.24 ± 0.21 nm). In both cases, uniform sizes were observed and no particular symmetries were observed. The size difference could be due to the fact that the HAuCl_4_ solution obtained from the coatings had a lower concentration than the Aldrich solution.

The particle size obtained with the HAuCl_4_ synthesized in this work is very similar to that reported by Turkevich, and by other authors [20], who used this synthesis method or some variant and obtained particle sizes between 10 to 20 nm. With other methodologies such as Brust–Schiffrin, synthesis with NaBH_4_ with/without citrate, synthesis with ascorbic acid, Seeding-Growth, and Green synthesis, it is possible to obtain similar particle sizes by modifying the concentration of the precursors [20]. Although other synthesis methodologies were not tested in the present work, the purity of the HAuCl_4_ obtained is sufficient to use in these methodologies.

These results indicate that HAuCl_4_ obtained from gold coatings recovered from e-waste processors has the purity required for use in nanoparticle synthesis, as the sizes and other properties are similar to those observed in Aldrich Au nanoparticles.

## 4. Conclusions

It is possible to dissolve 2 mg of Au coatings recovered from Intel Pentium-4 processors using only 10 mL of HCl 10 % *v*/*v* (1.21 mol L^−1^) and 50 μL of HNO_3conc_ (0.08 mol L^−1^) by a conventional heating method. The size and shape of the gold affect the conditions for dissolution of gold coatings. The low amounts of acids used has several advantages over the methods with aqua regia, chlorine gas and HNO_3_ with different salts mentioned above, e.g., (1) zero NO_2_(g) emissions due to H_2_O_2_ traps, which allows the recovery of HNO_3_; (2) easy separation of gold coatings due to its density; (3) the possible use of Cu(NO_3_)_2_ in other processes; (4) the recovery of Sn as an oxide, which can be used as a semiconductor; and (5) this process does not generate waste. In addition, the HAuCl_4_ synthesized in this work shows the purity necessary for the synthesis of gold nanoparticles, with sizes and shapes similar to those obtained with Aldrich HAuCl_4_, which could contribute to the development of this area.

Although the method proposed in this work requires further studies for its industrial application, the results obtained can serve as a basis for developing a better e-waste recycling process.

## Figures and Tables

**Table 1:** Chemical composition of the single pin by EDS analysis of the inner body, gold coating and base. Standard deviation is shown in parentheses.

Elements/Inner Body	Wt%
Cu	96.05 (2.98)
Ni *	1.72 (0.00)
Fe	2.23 (0.04)
**Elements/gold coating**	**Wt%**
Au	100 (0.0)
**Elements/base**	**Wt%**
Sn	100 (0.0)

* Detected in one point near the surface.

**Table 2:** Chemical composition by MP-AES analysis of the digestion of 478 pins with aqua regia.

Element	Au	Ni	Fe	Cu
Mass contained (g)	0.0199	0.1050	0.0019	2.0611
Percent (Wt%)	0.9	4.8	0.1	94.2

**Table 3:** Mass and MP-AES efficiencies of the products obtained in batches 1, 2 and 3.

Variables	Batch 1	Batch 2	Batch 3
Pins mass (g)	2.5119 (0.0004)	2.5392 (0.0004)	2.6118 (0.0002)
Au recovered (g)	0.0164 (0.0002)	0.0151 (0.0002)	0.0204 (0.0002)
Cu recovered (g)	1.9318 (0.0004)	1.7865 (0.0002)	1.6611 (0.0002)
Sn recovered (g)	0.4834 (0.0008)	0.0976 (0.0002)	0.2485 (0.0004)
m_T_ recovered (g)	2.4316 (0.0008)	1.8992 (0.0004)	1.9300 (0.0005)
m_T_ efficiency (%)	96.80 (1.48)	74.79 (1.24)	73.90 (0.72)
MP-AES efficiency * (%)	89.05 (0.02)	82.35 (0.02)	76.86 (0.02)

* Only Au and Cu masses and their uncertainties were considered [27].

**Table 4:** Concentration of Au, Ni and Cu obtained by MP-AES, and concentration of HAuCl4 obtained by the calibration curve of products 1.1 and 1.2.

Variables	Synthesis 1.1	Synthesis 1.2
[HAuCl_4_] (mM) *	1.04 (0.03)	0.96 (0.03)
Au dissolved percent (%)	101.9 (17.1)	105.2 (21.1)
[Au]_MP-AES_ (mM) *	1.04	0.95
[Ni]_MP-AES_	0.08 μM * (0.48 ppm)	0.12 μM * (0.73 ppm)
[Cu]_MP-AES_	0.32μM * (2.03 ppm)	0.87 μM * (5.51 ppm)

* mM = mol L^−1^ × 10^−3^ and μM = mol L^−1^ × 10^−6^.

**Table 5:** Chemical composition by MP-AES analysis of the HAuCl_4_ before and after the evaporation cycles.

Element	Au	Ni	Fe	Cu
Concentration before	1.07 mM *	ND	ND	0.25 ppm
Concentration after	0.63 mM *	ND	2.5 ppm	1.25 ppm

* mM = mol L^−1^ × 10^−3^.

## Data Availability

The data presented in this study are available on request from the corresponding author.
